# Working Memory and Reinforcement Schedule Jointly Determine Reinforcement Learning in Children: Potential Implications for Behavioral Parent Training

**DOI:** 10.3389/fpsyg.2018.00394

**Published:** 2018-03-28

**Authors:** Elien Segers, Tom Beckers, Hilde Geurts, Laurence Claes, Marina Danckaerts, Saskia van der Oord

**Affiliations:** ^1^Research Unit Behaviour, Health and Psychopathology, KU Leuven, Leuven, Belgium; ^2^Research Unit Brain and Cognition, University of Amsterdam, Amsterdam, Netherlands; ^3^Faculty of Medicine and Mental Health (CAPRI), University of Antwerp, Antwerp, Belgium; ^4^Department of Neurosciences, Child and Adolescent Psychiatry, University Hospital Leuven, KU Leuven, Leuven, Belgium; ^5^Developmental Psychology, University of Amsterdam, Amsterdam, Netherlands

**Keywords:** BPT, PREE, working memory, acquisition, extinction

## Abstract

**Introduction:** Behavioral Parent Training (BPT) is often provided for childhood psychiatric disorders. These disorders have been shown to be associated with working memory impairments. BPT is based on operant learning principles, yet how operant principles shape behavior (through the partial reinforcement (PRF) extinction effect, i.e., greater resistance to extinction that is created when behavior is reinforced partially rather than continuously) and the potential role of working memory therein is scarcely studied in children. This study explored the PRF extinction effect and the role of working memory therein using experimental tasks in typically developing children.

**Methods:** Ninety-seven children (age 6–10) completed a working memory task and an operant learning task, in which children acquired a response-sequence rule under either continuous or PRF (120 trials), followed by an extinction phase (80 trials). Data of 88 children were used for analysis.

**Results:** The PRF extinction effect was confirmed: We observed slower acquisition and extinction in the PRF condition as compared to the continuous reinforcement (CRF) condition. Working memory was negatively related to acquisition but not extinction performance.

**Conclusion:** Both reinforcement contingencies and working memory relate to acquisition performance. Potential implications for BPT are that decreasing working memory load may enhance the chance of optimally learning through reinforcement.

## Introduction

For childhood externalizing behavior various forms of behavioral parent training (BPT) have been established as first-line, evidence-based treatment (e.g., Chorpita et al., 2002; Evans et al., 2014). These interventions, which are generally developed to improve the parent-child relationships and reduce the child's misbehavior, are mainly based on behavior management principles derived from social learning theory and principles of operant learning (Zwi et al., 2011; Furlong et al., 2012). While different BPT programs have been developed, they all share common characteristics (Kazdin, 1997; Mash and Johnston, 2008). The underlying idea is that the child's behavior is influenced by its antecedents and consequences and that parents can learn to effectively modify these antecedents and consequences (Kazdin, 1997). By giving clear directions, positively reinforcing appropriate behavior and/or punishing inappropriate/unwanted behavior, parents can maximize the child's compliance and minimize disruptive behavior (Mash and Johnston, 2008; Zwi et al., 2011). Although most BPT programs are moderately efficacious as a package (Weisz and Kazdin, 2010), more research is needed on the individual and relative efficacy of distinct components of these training programs (Kaminski et al., 2008; Leijten et al., 2015). Moreover, some of the fundamental principles of BPT interventions have hardly been studied experimentally through basic operant learning paradigms in children. Yet enhanced experimental knowledge may give valuable information for treatment development and adaptation (Emmelkamp et al., 2014).

BPT programs emphasize the importance of positive and contingent reinforcement to reduce disruptive behavior in children (Leijten et al., 2015). A relevant but relatively unexplored phenomenon in children in this context is the Partial Reinforcement Extinction Effect (PREE; Skinner, 1938; Humphreys, 1939). The PREE is a paradoxical reward effect that illustrates how subtle features of reinforcement schedules can influence the acquisition and persistence of instrumental behavior. In operant conditioning, different schedules of reinforcement can be used. A schedule of continuous reinforcement (CRF) involves a 100% contingency between behavior and reinforce: Every correct response is reinforced. A schedule of partial reinforcement (PRF) involves a contingency of less than 100% between behavior and reinforcer. The PREE pertains to the observation that the schedule of reinforcement that was in operation when a behavior was acquired will critically determine the subsequent persistence of that behavior under extinction (i.e., when reinforcement is no longer provided; Domjan and Burkhard, 1993; Shangha et al., 2002). When behavior is reinforced partially or intermittently rather than continuously during acquisition, extinction occurs much more slowly once reinforcement is ceased, implying that a higher level of behavioral persistence has been created (Pittenger, 2002; Domjan, 2005). Moreover, research in animals and human adults suggests that repeated experience with PRF of desired behavior can lead to generalized behavioral persistence in the face of setbacks, adversity and lack of (further) reinforcement (e.g., McCuller et al., 1976; Boyagian and Nation, 1981; for a review see Amsel, 1992).

The PREE appears robust and replicable in animals (e.g., Lilliquist et al., 1999; Rescorla, 1999; Sangha et al., 2002; Gómez et al., 2008). Moreover, although human performance to specific experimental variables is not always consistent with animal study results, research suggests that humans and animals respond mostly in the same way to PRF (Pittenger et al., 1988; Pittenger, 2002). Research on the PREE in humans is largely limited to adults; research in children is scarce and methodologically very diverse. Findings in adults cannot necessarily be transposed to children and the few existing studies in children are hard to compare due to methodological differences (e.g., Bijou, 1957; Miyao and Meyers, 1973; Pittenger, 2002; Svartdal, 2003). Additionally, studies investigating the PREE have mainly explored the effects of partial vs. CRF on response extinction and not the differential effect of CRF and PRF on acquisition performance.

Studies of operant learning under PRF in adult humans have yielded divergent results. Some studies found no differential effect of CRF and PRF on acquisition performance, whereas other studies found PRF to interfere with the acquisition of an operant response, effectively preventing learning (e.g., Pittenger and Pavlik, 1988; Svartdal, 2003). A possible explanation for these divergent results is that human operant learning relies on executive functions (EFs) such as working memory (Capaldi, 1994; Collins and Frank, 2012). Working memory, defined as a limited capacity system allowing to temporarily hold and manipulate information “on-line” (Baddeley and Della Sala, 1996), is crucially involved in response acquisition, even in simple learning tasks. Collins and Frank (2012) demonstrated that learning was slower when the task implied a greater WM load, although only minimal differences in end-level performance were found. Powell et al. (2005) hypothesized that WM capacity is needed to carry the memory trace across unreinforced trials so that learning can take place. Especially PRF may place severe constraints on working memory and the larger the cognitive demands, the less likely it is that conditioning will occur (McKell Carter et al., 2002; Powell et al., 2005).

As BPT is often used for various childhood disorders (e.g., ADHD, autism, disruptive behavior disorders) in which WM capacity is assumed to be impaired (Martinussen et al., 2005; Barch and Ceaser, 2012; Barendse et al., 2013; Saarinen et al., 2015), it is vital to know to what extent WM deficits relate to the capacity for learning through reinforcement in children. Moreover, given the importance of PRF for the acquisition of behavioral persistence and the fact that PRF is a more feasible model for everyday parental reinforcement than CRF, it is important to evaluate whether possible WM-related deficits in learning trough reinforcement might be exacerbated under PRF.

In summary, BPT, often used to handle childhood disorders associated with working memory problems, is based on operant learning principles. Despite the strong theoretical underpinnings, the translation from theory to discrete parenting techniques seems to be based on expert clinical judgment and is rarely tested empirically in children (Leijten et al., 2015). Furthermore, studies investigating PREE are mainly interested in response extinction and do not focus on the effect of PRF vs. CRF on response acquisition nor do they explore the role of WM. Given that in daily life, reacting to the behavior of a child in a contingent and consistent way is the exception rather than the rule, we want to take a closer look at the difference between PRF and CRF in shaping the acquisition of behavior and at the role of WM therein.

In this study, children aged 6–10 years old (the typical age range for which BPT is provided) performed an operant learning task, in which a response-sequence rule had to be acquired based on feedback (CRF or PRF), followed by an extinction phase. Afterwards, their visuospatial WM was assessed. We investigated the influence of different reinforcement contingencies (CRF and PRF) on response acquisition and extinction. We hypothesized that acquisition would proceed faster under CRF than under PRF (hypothesis 1) but that behavior would persist longer in extinction after PRF than CRF (hypothesis 2). We also evaluated how WM capacity relates to those processes. As the interval between reinforcement is larger in PRF and a hypothetical response-sequence rule has to be kept in mind longer in PRF (thus putting more load on WM), we expected learning under PRF to correlate more strongly with WM capacity than learning under CRF (hypothesis 3). Although animal studies suggest that the same higher-level brain functions may be involved in extinction and WM (Callaerts-Vegh et al., 2006), no studies have specifically investigated the role of WM in extinction learning. Therefore, no specific hypotheses were formulated concerning the relation between WM capacity and extinction after PRF or CRF.

## Method

### Participants

Ninety-seven children (43 boys, 54 girls) in the age range from 6 to 10 years participated. Children were recruited through elementary schools and youth movements. Eligibility was determined by the following criteria: (a) absence of any parent-reported diagnosis, as classified by the fourth edition of the Diagnostic and Statistical Manual of Mental Disorders (DSM-IV-TR; American Psychiatric Association, 2000), (b) absence of any neurological disorder, sensory or motor impairment, as reported by the parents, (c) not taking any medication that could cause behavioral changes and/or influence attention, and (d) a total IQ score ≥ 80, to ensure that all children had the intellectual capacities to understand the tasks and to make sure that WM performance and learning performance were not mainly influenced by general cognitive capacities. IQ was estimated using the short version of the Dutch Wechsler Intelligence Scale for Children (WISC-III-NL; Kort et al., 2002). Full Scale IQ (FSIQ) was estimated based on the subtests Vocabulary and Block Design. The combined score of these two subtests has a satisfactory reliability (*r* = 0.91) and correlates highly with FSIQ (*r* = 0.86; Sattler, 2001). A total of 88 children (42 boys, 46 girls) met the inclusion criteria. One child did not want to proceed during the operant learning task and dropped out during testing, leaving a final sample of 87 children (42 boys, 45 girls; 48.3% male). All participants were Caucasian. Randomization to the PRF or CRF condition was stratified on age and gender. CRF and PRF groups did not differ with respect to age, gender, FSIQ, WM performance (see Table 1) and scores on the Child Behavior Checklist for ages 6–18 (CBCL 6–18; Achenbach and Rescorla, 2001; Dutch version: Verhulst et al., 1996; see Table 2). The CBCL was administered to assess general level of behavioral and emotional problems and to check whether groups were comparable on these variables.

**Table 1:** Means and standard deviations of group demographics and characteristics.

**Measure**	**CRF group *n* = 39)**		**PRF group *n* = 48)**		***F*/χ^2^**	***p***
	***M***	***SD***	***M***	***SD***		
Gender (M:F)	18:21	–	24:24	–	0.013	0.721
Age in years	8.58	1.47	8.56	1.45	0.03	0.956
Estimated FSIQ	104.74	15.93	108.02	21.08	0.64	0.425
WM performance	4.80	0.99	4.96	1.03	0.54	0.46

*CRF, continuous reinforcement; PRF, partial reinforcement; FSIQ, full scale IQ; M:F, male: female; WM performance, working memory performance*.

**Table 2:** Means and standard deviations for subscales of the Child Behavioral Checklist (mother report).

**Measure**	**CRF group (*n* = 39)**		**PRF group (*n* = 48)**		***F***	***p***
	***M***	***SD***	***M***	***SD***		
Withdrawn/depressed	0.46	0.72	0.65	1.04	0.44	0.51
Anxious/depressed	2.28	2.50	1.92	2.59	0.88	0.35
Somatic complaints	0.95	1.38	0.96	1.09	0.001	0.97
Aggressive behavior	3.13	4.90	2.31	2.83	0.95	0.33
Rule-breaking behavior	1.15	1.63	0.65	0.96	3.28	0.07
Thought problems	1.59	2.04	1.69	2.10	0.048	0.83
Attention problems	1.92	2.49	2.50	2.67	1.07	0.31
Social problems	1.36	2.15	1.15	2.04	0.22	0.64
Other problems	2.31	2.20	2.15	2.38	0.11	0.75
Internalizing problem cluster	3.69	3.76	3.52	3.91	0.034	0.84
Externalizing problem cluster	4.28	6.35	2.96	3.46	1.53	0.22
Total problem scale	15.33	15.37	13.96	12.62	0.21	0.35

*CRF, continuous reinforcement; PRF, partial reinforcement*.

### Procedure

The study procedure and materials used were approved by the Ethical Committee of the Faculty of Psychology and Educational Sciences of the KU Leuven. Prior to testing, parents filled out an informed consent form and three questionnaires (i.e., the CBCL 6–18 and two other questionnaires that were administered for another study). The children were then tested individually in a laboratory setting. A learning task, a WM task and an inhibition task were administered. The inhibition task (stop-signal paradigm; Logan, 1994) was administered for purposes unrelated to the present study. The test sessions always started with the learning task to avoid carry-over effects of the other tasks on performance (e.g., strategy use). After the learning task, a 15 min break was inserted in which the child had a drink and played in a designated play-area, to avoid potential fatigue. Subsequently, the WM and inhibition task were administered in counterbalanced order^1^. Finally, the IQ subtests were administered. Participants received a small gift at the end of the session.

### Tasks and questionnaires

#### Operant learning task

**Figure 1:**
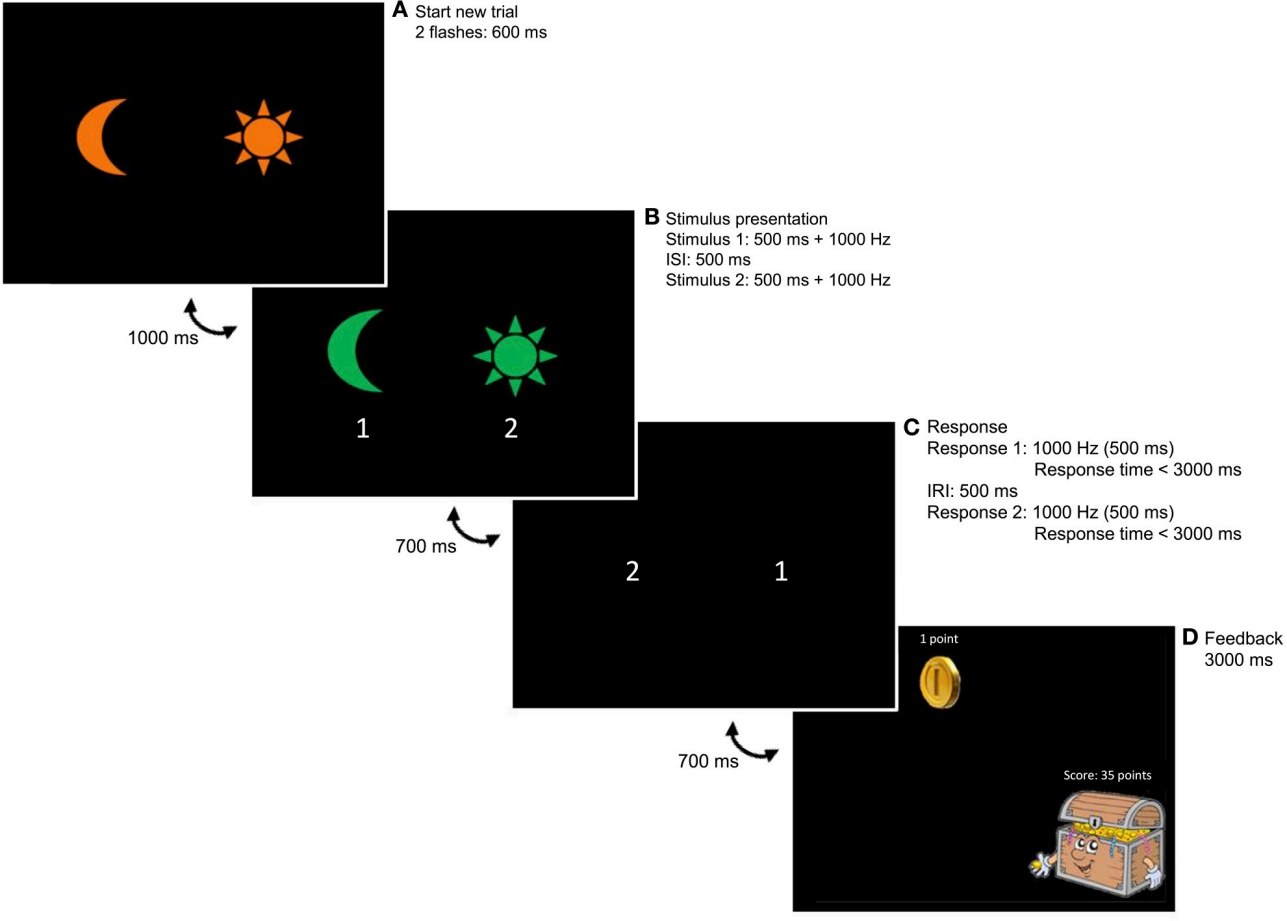
Example trial of the learning task. **(A)** Two orange stimuli simultaneously flash twice, indicating the start of a new trial. **(B)** Two green stimuli appear. In this example, the moon appears first. When the moon has disappeared, the sun appears on the screen. **(C)** A first tone prompts a first response, followed by the second tone prompting a second response. Correct responding occurs when the stimulus sequence is reversed. In this example, a correct response is given when the participant presses the sun button (right) after the first prompt and the moon button (left) after the second prompt. **(D)** Upon responding correctly, CRF participants always received reinforcement; PRF participants received reinforcement on 60% of correct trials.

The operant learning task was a newly developed task, based on a learning task of Svartdal (2003). Participants were instructed to learn and apply a response-sequence rule based on feedback when responding correctly. Two stimuli were used, a picture of a moon and a sun. These were always presented at the left and right side of the computer screen, respectively. To signal the start of a trial, an orange version of the moon and sun were presented simultaneously on the computer screen, which flashed two times for 600 ms. After a 1,000 ms pause, a green version of the moon and sun were presented one by one, each lasting for 500 ms, accompanied by a 1,000 Hz tone and with a 500 ms pause in between. After another 700 ms pause, the child's first response was prompted by a 1,000 Hz tone, followed by the second response prompt after a 500 ms pause. The maximum time to respond was 3,000 ms after each prompt (for a visual presentation of the task, see Figure 1). Participants had to respond by means of two marked buttons on the keyboard (i.e., moon left and sun right).

The response-outcome contingency was always related to the position of the stimuli presented: participants responded correctly when they reversed the sequence of the two computer stimuli within the same trial. For example, when the sun (right) appeared first on the screen, followed by the moon (left), the correct response was to press the moon button first (left), followed by the sun button (right). The four different stimulus combinations [i.e., (1) sun (2) sun; (1) sun (2) moon; (1) moon (2) moon; (1) moon (2) sun] were presented at random. Each combination was presented equally often (i.e., 30 times in the acquisition phase and 20 times in the extinction phase). Correct sequences were followed by feedback dependent on the reinforcement condition. Feedback for correct responding consisted of a coin, which appeared in the middle of the screen for 900 ms, followed by the sound of falling coins for 600 ms and a treasure chest displaying the total amount of coins earned so far. Participants were instructed to obtain as many feedback messages (i.e., coins) as possible (instructions in Appendix).

To familiarize the children with the task, the experimenter first demonstrated the task for 10 trials, after which the child performed 16 practice trials during which no feedback was provided. Participants were randomly assigned (between subjects) to the CRF condition (i.e., 100% probability of reinforcement for correct responding) or PRF condition (i.e., 60% probability of reinforcement for correct responding). So, in the 120 trials of the acquisition phase, children in the CRF condition always earned a coin when giving a correct response, whereas children in the PRF condition earned a coin for 60% of their correct responses only (i.e., 6 randomly delivered reinforcements per 10 correct responses). In the 80 trials of the extinction phase, feedback was ceased. At the end of the task, the total number of coins appeared on the screen. The percentage of correct responses during the acquisition phase and the extinction phase was the primary dependent measure.

#### Working memory task

The Chessboard Working Memory Task is a visuospatial working memory task (Dovis et al., 2012) and is based on the Corsi Block Tapping Task (Corsi, 1972) and the subtest Letter-Number Sequencing from the Wechsler Adult Intelligence Scale (Wechsler, 1958). It assesses the ability to hold and manipulate/reproduce visuospatial information and is individually adapted to performance during the task to test the child's optimal working memory performance (Dovis et al., 2012).

A 4 × 4 grid consisting of green and blue squares (stimuli) in a chessboard formation was presented. A number of squares then lighted up in sequence for 900 ms each, accompanied by a brief tone presented through headphones and separated by an inter-stimulus-interval of 500 ms. After the sequence was presented, the participant had to reorganize the sequence by mouse-clicking on the squares in the following way: the green stimuli had to be reproduced before reproducing the blue stimuli, each in the same sequence as they were presented. To ensure that every presented sequence had to be reorganized, the order of the stimuli was randomized with the restriction of presenting at least one blue stimulus before the last green stimulus in every sequence.

The difficulty of the task was adapted to the performance of the child. The minimum sequence length consisted of two stimuli. After two successive correct reproductions, the sequence was lengthened with one stimulus. After two successive incorrect reproductions, the sequence was reduced with one stimulus. As the task was individually adapted, the amount of positive and negative feedback was approximately the same for each participant (55% positive, 45% negative feedback). After each trial, feedback was given both in case of correct responding (i.e., a green curl and a positive guitar sound) and in case of incorrect responding (i.e., a red cross and a negative buzzer sound).

The task started with a practice block (5 trials) followed by an experimental block (30 trials) and took about 20 min. WM performance was measured by the mean sequence length on the last 18 trials, as the first 12 trials of the task are needed to reach the child's optimal difficulty level (Dovis et al., 2012).

#### Child behavior checklist for ages 6–18 (CBCL 6–18)

The Child Behavior Checklist for ages 6–18 (Achenbach and Rescorla, 2001; Dutch Version: Verhulst et al., 1996) is a standardized parent-rated questionnaire measuring internalizing and externalizing problems of children. The questionnaire has adequate reliability and validity (Achenbach and Rescorla, 2001). One hundred thirteen questions are rated on a 3-point Likert scale, measuring eight symptom subscales of internalizing and externalizing behavior.

## Data analysis

First, it was tested whether participants produced more correct responses during acquisition than extinction and whether there was a difference between reinforcement conditions in this ratio (indicative of a PREE), through two repeated-measures ANOVAs. In the first analysis, the percentage of correct responses was analyzed using reinforcement condition (CRF/PRF) as between-subject factor and phase (acquisition vs. extinction) as within-subject factor. In a second analysis, the number of correct responses per block of 10 trials was evaluated with reinforcement condition (CRF/PRF) as between-subject factor and trial block (1–20) as within-subject factor.

To evaluate the influence of different reinforcement contingencies on response acquisition separately (hypothesis 1), an ANOVA was then conducted with reinforcement condition (CRF, PRF) as between-subject factor and the percentage of correct responses during the acquisition phase as dependent variable. To probe whether WM was related to response acquisition (hypothesis 3), a repeated-measures ANCOVA was performed with reinforcement condition (CRF, PRF) as between-subject factor, WM performance as covariate and the percentage of correct responses during acquisition as dependent variable.

Analyses of the extinction data were similar to those of the acquisition data, be it that terminal acquisition performance (i.e., the percentage of correct responses over the last three trial blocks of acquisition) was included as a covariate in the extinction analysis to control for differences in asymptotic acquisition performance. Thus, to assess the influence of different reinforcement contingencies on extinction (hypothesis 2), an ANCOVA was performed with reinforcement condition (CRF, PRF) as between-subject factor, end level of acquisition as covariate, and the percentage of correct responses during extinction as dependent variable. To check whether WM was associated with extinction performance, an ANCOVA was conducted with reinforcement condition (CRF, PRF) as between-subject factor, WM performance and terminal acquisition performance as covariates, and percentage of correct responses in extinction as dependent variable.

Estimated effect sizes are reported for all analyses (partial η^2^); η^2^_*p*_ = 0.01 is regarded a small, 0.06 a medium, and 0.14 a large effect size (Kittler et al., 2007).

## Results

**Figure 2:**
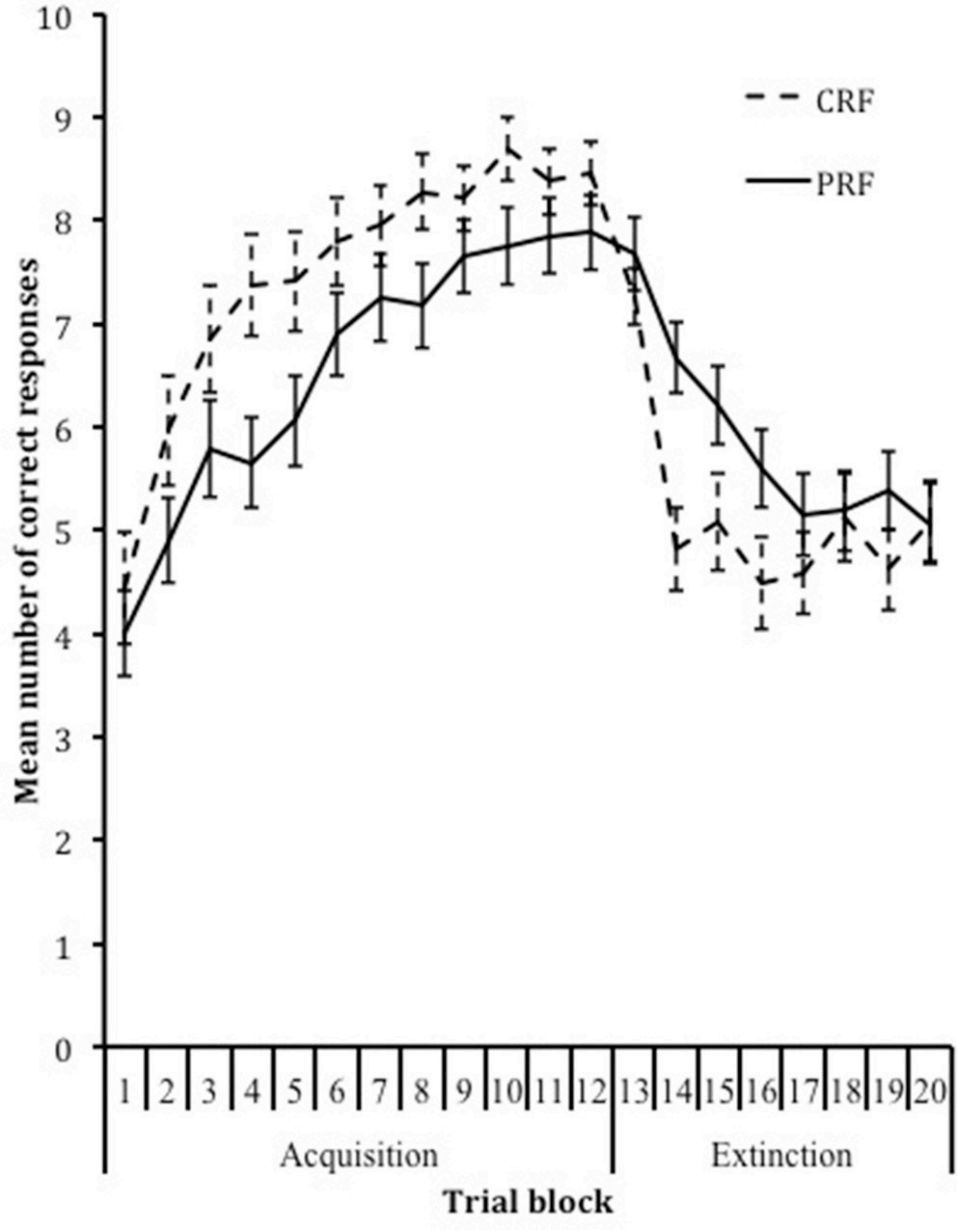
Acquisition and extinction of the response over trial blocks in the CRF and PRF conditions.

To test whether a PREE was obtained, a 2 × 2 (Phase × Reinforcement Condition) repeated-measures ANOVA was conducted on the percentage of correct responses. This analysis showed a significant main effect for phase, *F*_(1, 85)_ = 50.23, *p* < 0.001, η_*p*_^2^ = 0.37. As expected, the percentage of rule-based responses was significantly higher during the acquisition phase (*M* = 69.80, *SD* = 20.52) compared to the extinction phase (*M* = 55.36, *SD* = 20.77). Furthermore, a significant interaction effect was found, *F*_(1, 85)_ = 14.60, *p* < 0.001, η_*p*_^2^ = 0.15. As expected, compared to partially reinforced children, continuously reinforced children produced a higher percentage of correct responses during the acquisition phase (CRF: *M* = 74.85, *SD* = 20.06; PRF: *M* = 65.69, *SD* = 20.17) and a lower percentage during the extinction phase (CRF: *M* = 51.31, *SD* = 20.22; PRF: *M* = 58.65, *SD* = 20.83). Additionally, a 20 × 2 (Trial Block × Reinforcement Condition) repeated-measures ANOVA showed a significant linear, *F*_(1, 85)_ = 9.04, *p* = 0.003, η_*p*_^2^ = 0.096, quadratic, *F*_(1, 85)_ = 174.91, *p* < 0.001, η_*p*_^2^ = 0.67, and cubic, *F*_(1, 85)_ = 52.35, *p* < 0.001, η_*p*_^2^ = 0.38, main effect for trial block and a cubic interaction effect, *F*_(1, 85)_ = 14.02, *p* < 0.001, η_*p*_^2^ = 0.14, implying that continuously reinforced children learned the response-sequence rule faster during acquisition and showed faster extinction of this previously learned rule during extinction than partially reinforced children (Figure 2), confirming the PREE.

### Acquisition

An ANOVA on the acquisition phase separately confirmed that children in the CRF group had a higher percentage of correct responses during acquisition than those in the PRF group, *F*_(1, 85)_ = 4.46, *p* = 0.038, η_*p*_^2^ = 0.050.

Analysis of covariance (with working memory as covariate) on the percentage of correct responses showed that not only the reinforcement condition, *F*_(1, 84)_ = 6.20, *p* = 0.015, η_*p*_^2^ = 0.069 (more correct responses under CRF than under PRF), but also WM capacity, *F*_(1, 84)_ = 11.08, *p* = 0.001, η_*p*_^2^ = 0.12, related to response acquisition (lower WM predicted less correct responses). Additional *post-hoc* correlational analysis suggested that the correlation between working memory performance and the percentage of correct responses in acquisition was significant in the CRF group but not in the PRF group [CRF: *r*_(39)_ = 0.59, *p* < 0.001; PRF: *r*_(48)_ = 0.34, *p* = 0.28]. However, a Fisher *r*-to-*z* transformation showed no significant difference between those two correlation coefficients (*z* = 1.45, *p* = 0.147).

Summarized, results show that continuously reinforced children acquired the response-sequence rule faster than partially reinforced children and that, independent of reinforcement condition, WM capacity was positively related to learning performance.

### Extinction

An ANCOVA (controlling for terminal acquisition performance) on the percentage of correct responses in the extinction phase showed significant effects of reinforcement condition, *F*_(1, 84)_ = 7.45, *p* = 0.008, η_*p*_^2^ = 0.082 (lower percentage of correct responses in extinction after CRF than PRF), and performance in the last 3 blocks of acquisition, *F*_(1, 84)_ = 27.67, *p* < 0.001, η_*p*_^2^ = 0.25 (higher terminal acquisition performance was associated with higher percentage of correct responses during extinction) (hypothesis 2). Analysis of covariance with WM and terminal acquisition performance as covariates showed a significant effects of reinforcement condition, *F*_(1, 83)_ = 6.84, *p* = 0.011, η_*p*_^2^ = 0.076, and terminal acquisition performance, *F*_(1, 83)_ = 23.54, *p* < 0.001, η_*p*_^2^ = 0.22, but no effect of WM, *F*_(1, 83)_ = 0.42, *p* = 0.52, η_*p*_^2^ = 0.005.

Summarized, results show that extinction of the response-sequence rule took place faster after CRF than after PRF. WM was not related to the speed or extent of extinction.

## Discussion

The purpose of this study was to investigate the differential effect of CRF and PRF on response acquisition and extinction and to evaluate whether WM is implicated in simple operant learning performance. Most of our predictions were confirmed. As predicted (based on studies in animals and human adults; Sangha et al., 2002; Svartdal, 2003), continuously reinforced children produced a higher rate of correct responses during acquisition than partially reinforced children and reached higher terminal levels of correct responding (hypothesis 1).

As several studies showed that human operant learning involves WM and on the basis of the idea that PRF places stronger demands on WM, we hypothesized that WM plays a role in response acquisition in general and that PRF loads more on WM than CRF when acquiring new behavior because reinforcement contingencies have to be bridged over a longer time period in PRF (Capaldi, 1994; Collins and Frank, 2012). Our results showed that WM capacity was positively associated with acquisition performance, independent of the reinforcement condition (hypothesis 3). This mirrors other studies that show WM involvement in associative learning, even in simple learning tasks (McKell Carter et al., 2002; Powell et al., 2005). Contrary to our prediction, WM was not associated more strongly with PRF learning than with CRF learning.

In line with the consistent observation of a PREE in animals and human adults (e.g., Pittenger et al., 1988; Lilliquist et al., 1999; Rescorla, 1999; Pittenger, 2002; Sangha et al., 2002; Gómez et al., 2008), we predicted that previously partially reinforced children would persist longer during extinction compared to previously continuously reinforced children (hypothesis 2). This hypothesis was confirmed in our results; the decline of previously reinforced responding occurred faster under CRF than PRF, suggesting a greater level of behavioral persistence when behavior is reinforced intermittently rather than consistently. Results showed no association between WM capacity and extinction performance. Extinction seemed to be determined mainly by the reinforcement schedule in place during acquisition (CRF vs. PRF), independent of WM capacity.

From these results, two potential clinical implications can be derived. A first implication has to do with the finding that not only the contingency of reinforcement but also WM capacity is critically associated with the capacity to acquire new behavior. BPT, which is based on operant learning principles, is often used for various childhood disorders in which WM is assumed as an underlying cognitive deficit. Pending conformation that our task paradigm has ecological validity for children's responding to operant contingencies provided by parents in daily life and that the relations observed here can be generalized to clinical samples, our results tentatively suggest that the children at which BPT is directed are exactly the ones that may be least susceptible to BPT interventions, because their WM deficits could place them at a disadvantage for learning through reinforcement. Perhaps then, those WM deficits could also be a target for intervention. One way to potentially enhance the effectiveness of BPT for those populations may be to first improve WM performance through decreasing WM load. In some BPT programs (but not all, see Chorpita and Daleiden, 2009) antecedent techniques are first implemented, aimed at unburdening working memory, before implementing operant techniques. Our results support the implicit tenets of these BPTs, as the release of working memory resources should increase the chance of optimally learning through reinforcement and decrease the possible influence of working memory deficits. However, given that clinical samples are quite heterogeneous with regards to WM capacity (Dovis et al., 2015), assessment and tailoring of BPT toward individual WM performance may be essential. Also, as our study suggests that individual differences in WM capacity may be associated with the response to BPT, this neurocognitive factor may be a more proximal and mechanistically relevant candidate to explore in moderation research of BPT then the more distal environmental moderators (e.g., SES) that are typically explored (e.g., Matthys et al., 2012).

A second implication is related to the persistence of new, desirable behavior in children. Our results suggest that children learn new behavior faster and more strongly under CRF than PRF. However, in daily life, behaving in a contingent and consistent way toward a child (i.e., reinforcing every instance of desired behavior) is the exception rather than the rule (Catania, 2013). Moreover, our extinction findings suggest that in order to create behavioral persistence, PRF is to be preferred over CRF. This represents somewhat of a paradox: The most feasible parental reinforcement method, which is also likely to yield the strongest persistence of desired behavior, is actually least suited for the acquisition of desired behavior. One way out for this conundrum may be a strategy of *stretching the ratios* when shaping desired new ways of behaving. The general idea here is to initially reinforce the child continuously when behaving in a correct way (i.e., emitting a desired response; CRF) and to then very gradually reduce the rate of reinforcement (PRF) (Skinner, 1968). Animal research suggests that this strategy can promote behavioral persistence of appropriate behavior to a similar extent as immediate PRF. Sangha et al. (2002) explored the effect of different contingency patterns on the ability to learn in snails. Their study showed that when CRF was followed by PRF, memory was resistant to extinction. Most BPT programs stress the importance of CRF in order to attain robust behavior change, and although some programs include stretching the ratios not all programs include this technique (Chorpita and Daleiden, 2009). Supplementing a CRF strategy with a gradual spacing out of reinforcement after the observation of initial behavior change might prove more effective in ensuring that behavior change persists. We should note, however, that our study did not include measures of frustration during PRF or extinction and that it was not conducted in a clinical sample. Following Amsel's frustration theory, especially in more clinical samples (where emotional dysregulation and irritability is generally higher, e.g., Brotman et al., 2017), the frustration experienced during PRF and extinction may interfere with the emergence of behavioral persistence (Nation and Woods, 1980; Amsel, 1992). As such, to determine the potential of stretching the ratios to enhance behavioral persistence after initial CRF, future research should test our procedure in clinical samples and include a stretching the ratios condition and measures of frustration.

The current study has some limitations that need to be discussed. First, the operant learning task consisted of 120 acquisition trials and 80 extinction trials. A pilot study showed that this amount of trials was needed to reach asymptotic response acquisition and extinction, however, it should be noted that because of the amount of trials the task was quite long for the children (approximately 40 min). The length of the task could possibly have had an effect on the attention span and/or accuracy, although there was no direct evidence for this during the study. Once the participants figured out the correct response, a steady response pattern was maintained. Performance did not show a decline of correct responding toward the end of the acquisition phase (Figure 2). A second limitation is that only typically developing Caucasian children with an IQ above 80 were included in this study, resulting in limited variability within the sample (e.g., in WM capacity). However, because research on response acquisition and extinction is scarce in children, we deemed it important to investigate these mechanisms in typically developing children first before studying these learning principles in different samples of children with mental health disorders. Further, one might argue that working memory performance is merely an indicator of intelligence and as such our results are simply a reflection of the role of intelligence in reinforcement learning. However, we found similar results when controlling for IQ, i.e., working memory remained significantly associated with acquisition performance, supporting the notion that our results are specific for working memory. Finally, to rule out potential carry-over effects (e.g., strategy use), the order of the learning and working memory tasks was not counterbalanced; the learning task was always presented first. Therefore, we cannot rule out that performance on the learning task influenced performance on the working memory task.

In conclusion, our study shows that CRF of desired responding results in faster and more robust acquisition than PRF in children and that, regardless of the continuous or partial nature of reinforcement, a higher WM capacity is associated with better learning. Extinction performance was determined only by whether children received PRF or CRF during acquisition and unrelated to WM capacity. This study is one of the first to investigate the effect of CRF and PRF on response acquisition and extinction in children; more studies are needed both in typically developing children and critically also in children with mental health problems to clarify reinforcement learning and its association to WM capacity in children. By doing so, further indications may be found for how to optimize therapeutic techniques such as BPT.

## Ethics statement

This study was carried out in accordance with the recommendations of the social and societal ethics committee of the KU Leuven with written informed consent from all subjects. All subjects gave written informed consent in accordance with the Declaration of Helsinki. The protocol was approved by the social and societal ethics committee of the KU Leuven.

## Author contributions

SvdO, TB, MD, ES, and HG designed the experiment and tasks. ES collected the data. ES, and LC analyzed the data, in close cooperation with TB, HG, and SvdO. ES drafted the manuscript, SvdO, TB, HG, MD, and LC provided extensive feedback.

### Conflict of interest statement

SvdO has been a paid speaker (Shire, MEDICE). Co-developer/author of a cognitive training game “Braingame Brian” and two cognitive-behavioral treatments “Plan my Life” and Solution-focused treatment (non-financial COI). MD has been a paid member of advisory boards organized by Shire, Novartis and Medice and paid for consultancy for Neurotech Solutions. She received research grants from Shire and Janssen-Cilag. The other authors declare that the research was conducted in the absence of any commercial or financial relationships that could be construed as a potential conflict of interest.

## Footnotes

^1^There was no significant difference in WM performance between children who completed the WM task before the inhibition task and the children who completed the inhibition task before the WM task. Additionally, there was no main effect of the order of those tasks on acquisition or extinction performance, nor was there an interaction between their order and reinforcement condition on acquisition or extinction performance (data available from corresponding author). Results from the inhibition task will not be presented.

## Appendix

### Instructions for the operant learning task (translated; original instructions in Dutch)

You will see two figures on the computer screen: a sun and a moon. First, these figures are orange and flash two times. This means that we will start. Afterwards, two figures will appear on the screen. These figures are always green. It is important to pay attention to the two figures, because afterwards you have to select two figures yourself on the keyboard. You can choose to press the button of the sun (experimenter points at the button of the sun) or the moon (experimenter points at the button of the moon). Which figures you have to select, you have to find out yourself. *Specific instructions CRF condition*: If you press the correct buttons, you will receive a coin. The more correct responses you give, the more coins you earn. The total amount of coins earned so far is indicated on the treasure chest. Please try to earn as much coins as possible. *Specific instructions PRF condition*: If you press the correct buttons, you will sometimes receive a coin and sometimes you will not. The more correct responses you give, the more coins you earn. The total amount of coins earned so far is indicated on the treasure chest. Please try to earn as much coins as possible.

## Abbreviations

BPT: behavioral parent training
CBCL 6-18: Child Behavior Checklist for ages 6–18
CRF: continuous reinforcement
DSM-IV-TR: Diagnostic and Statistical Manual of Mental Disorders (4th ed., text rev.)
EFs: Executive functions
FSIQ: full scale IQ
IQ: intelligence quotient
PBS: Parental Behavior Scale
PREE: partial reinforcement extinction effect
PRF: partial reinforcement
SSRT: stop signal reaction time
WISC-III-NL: Wechsler Intelligence Scale for Children
WM: working memory.
